# Special Issue “Molecular Research on Bacteria”

**DOI:** 10.3390/ijms27073043

**Published:** 2026-03-27

**Authors:** Giovanni Stelitano

**Affiliations:** Department of Biology and Biotechnology “L. Spallanzani”, University of Pavia, 27100 Pavia, Italy; giovanni.stelitano@unipv.it

Gaps in molecular research on bacteria remain that cover multiple areas, ranging from genetics to biochemistry and from basic to applied science, underscoring the need to address existing gaps at all levels.

This Special Issue collects recent findings that contribute to filling these gaps. For example, bacterial resistance and resilience to reactive oxidative species (ROS), as well as photodynamic stress, represent two distinct bacterial behaviors that share common molecular mechanisms resulting in similar phenotypes (Contribution 1). The possibility of discriminating between these behaviors may facilitate the design of combined and more effective therapeutic options.

Research continues to identify roles of genes and proteins that have remained unclear until now, such as genetic regulators’ role in cell metabolism and infection processes, or novel effectors involved in antibiotics and antimicrobial resistance mechanisms as well as host–pathogen interactions (Contributions 2–4). These processes are illustrated by the identification of at least 49 effectors expressed by *Salmonella* sp. during infection and their contribution to pathogenicity (Contribution 5). Another representative example is provided by the different expressions of homologous genes under the control of the same regulator depending on the environmental conditions. In *B. thuringiensis*, the *hemH1* and *hemH2* genes, both regulated by Fur, display a different expression pattern in the presence or absence of iron or under oxidative stress conditions (Contribution 6). Similarly, the pathway that drives the conversion of *N*^4^-methylcytosine into cytosine by bacteria such as *E. coli* is still uncertain, while recent studies are shedding new light on the molecular mechanisms and the selectivity of *N*^4^-methylcytosine rather than *N*^4^-*N*^4^-methylcytosine (Figure 1) (Contribution 7). Furthermore, recent research is consolidating the key role of plasmids in the spread of antibiotic resistance in the presence of a specific antibiotic concentration in the environment or at the site of infection during treatment (Contribution 8).

Genetic analyses are further deepening our understanding of bacterial phenotypic variability within the same cell population, addressing the gaps related to bacterial subpopulations and their differentiation (Contribution 9). Moreover, previous incorrect open reading frame (ORFs) annotations are being identified, which are estimated to affect approximately 7% of the entries in available databases. One such case involves *Escherichia coli*’s RatA protein, previously described as a ribosome-targeting toxin and recently validated as the bacterial homolog of the mitochondrial Coq10 protein, with a distinct role in cell metabolism (Contribution 3). Similar approaches are also instrumental in reconstructing bacterial evolutionary histories, from the environmental niches to pathogenic processes. An illustrative example involves environmental species that can produce photogenic toxins. Phylogenetic analyses indicated that *Enterococcus faecium*’s bile salt hydrolase BSH toxin may have evolved independently in environmental species before being transferred to pathogenic species through horizontal gene transfer (Contribution 10). Finally, genetic tools combined with other methodologies can uncover the drug susceptibility and resistance profiles of cross-species pathogens, such as *Staphylococcus saprophyticus*, enabling the rapid identification of the most appropriate therapeutic regimen. This bacterium, which commonly infects the human perianal region and genitourinary tract and shows resistance to penicillin in approximately 35% of cases, was recently found to be resistant to oxacillin in 42% of both human- and animal-infecting strains, as well as to erythromycin in all dog-infecting strains (Contribution 2).

Advances in basic knowledge are progressing alongside the growing need to develop new therapeutic options. In parallel with the discovery of new antibiotics and development of *de novo*-synthesized antimicrobials, phage cocktails are showing increased importance in terms of their ability to clear even the most difficult infection, including multidrug- and extensively drug-resistant strains (Contribution 11). Moreover, the development of novel and safer vaccines has the potential to alter disease outcomes at a scale by preventing infections, limiting pathogen spread, or even achieving complete disease eradication. This could be a winning strategy in eradicating typhoid salmonella cases (Contribution 12).

Overall, the contributions collected in this Special Issue highlight the remarkable complexity and adaptability of bacterial systems, spanning molecular mechanisms, genetic variability, and evolutionary dynamics. By integrating basic and applied research, these studies not only deepen our understanding of bacterial biology but also provide a solid foundation for the development of innovative and more effective therapeutic and preventive strategies. Together, they emphasize the importance of molecular research in addressing the current and emerging challenges posed by bacterial pathogens.

## Figures and Tables

**Figure 1:**
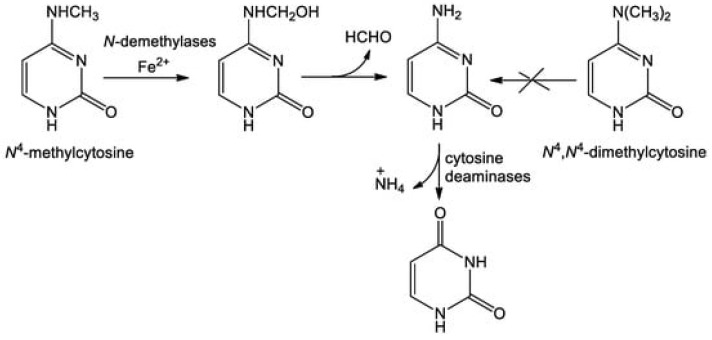
Proposed mechanism of the conversion of *N*^4^-methylcytosine into cytosine in *E. coli* (Contribution 7).

